# A Treatise on Irritation of the Spinal Nerves

**Published:** 1842-10

**Authors:** 


ART. VIII.—*A Treatise on Irritation of the Spinal Nerves as the source of Nervousness, Indigestion, functional and organic Derangements of the principal Organs of the Body, and on the modifying influence of Temperament and Habits of Man over Diseases, and their importance as regards conducting successfully the Treatment of the latter; and on the Therapeutic Use of Water.* By J. EVANS RIADORE, M.D. F.L.S. &c. &c.—*London*, 1842. 8vo, pp. 306.

ANYTHING more contemptible, more utterly devoid of interest and value, than the work, whose prolix title we have just copied, it has seldom been our misfortune to peruse. We trust our readers will give us due credit for a large share of patience, when we inform them that we have waded through the entire mass of rubbish, lest perchance any concealed jewel, worthy to be exposed to day, might lurk unseen: but all in vain; with the exception of sundry quotations from well-known and standard authorities, there is nothing in the whole 306 pages which is deserving of attention, much less of praise.

But while we find nothing to commend there is abundance to condemn. The author's neglect of grammar and ignorance of the very simplest rules of composition are no less marvellous as a fact than they are disgraceful in a member of a learned profession. We shall give a few specimens: "Some anatomists have considered that the twenty-four bones and joints forming the spinal column *is* too strongly fixed together to be dislocated." (p. 3.) "Pains in the abdomen and considerable distention of it by flatus *is* a common consequence." (p. 10.) "Extreme violence of treatment, *of* which there is a disposition to run *into*, in the present age," &c. (p. 45.) "This temperament when *unduly*, or in a morbid condition, is often increased by sedentary habits." (p. 57.) "The *bowels* of a patient of the nervous temperament *is* subject," &c. (p. 63.) "No inconvenient *symptoms follows*." (p. 76.) Enough for false syntax. The orthography is equally brilliant: The prostate gland is, with one single exception, universally spelt *prostrate*. *Scybelœ* and *scybela*, *morphin*, *lumber* for lumbar, *dyspectic*, *bicarbon of potash*, *sura scapula* (supra-scapular) nerve, *gangliœ*, *manupulation*, *sphinctars*, &c. diversify and adorn these luminous pages; and granting that some of them are mere typographical errors, what shall be said for the carelessness of an author who could overlook such glaring faults?

But even the plea of carelessness, miserable as it is, will scarcely hold good in all instances. Can it be from a mistake of the printer that "*alkaline*" is made a substantive? That we are informed (p. 56) that "*atrophy*, engrafted upon original asthenic or feeble constitution, *will generally die* in consequence of obstruction of the mesenteric glands?" That we hear of an infant "*suckling* a foster-mother?" (p. 26.) And that poor Sir Astley Cooper is said to have recommended "*suckling* to be protracted, as being more *nutricious* and *easier of digestion* than the food usually provided afterwards." (p. 66.)

In the prosecution of our wearisome task, we had marked numerous passages for quotation, as illustrative of the peculiarly lucid style which characterizes this most philosophical production; but we shall spare our readers the infliction, condemning them to the perusal of two only, which are selected because with them are associated the names of two very eminent individuals, who, we trust, will feel themselves duly honoured by Dr. Riadore's notice:

"The *laws* of sympathy, which have always been observed by practitioners, *is* in effect the theory arrived at and named by Dr. Hall *excito-motory system*, which he divided into two sets of nerves, the incident and reflex." (p. 118.)

"That the stomach and the urinary organs are frequently secondarily affected, and not, as Dr. Prout, in his work on Stomach and Urinary Diseases, attributes to malassimulation as the effect of primary cause engendered in the stomach to derange it and the kidneys functionally or organically, is rendered still more probable *if confirmed as a truth*, by the fact that indigestion is cured by applications to the spine, as well as affections of the kidneys," &c. (p. 230.)

Literary delinquencies of a character so flagrant as the above it was manifestly impossible to overlook; yet might we have touched them with a somewhat lighter hand, had the work been redeemed by anything in the slightest degree valuable in pathology or practice. That such is not the case is, however, but too certain. The one object of the author seems to be to establish his favorite theory, that almost every ill that flesh is heir to may be traced to that vague indefinite something, denominated "spinal irritation."

Dr. Riadore is strong in dietetics. "Our mode of thinking," he tells us, "and even *the form of religion and degree of attention paid to its rites*, are under the control of the diet!" And yet he says in another place that "drunkards generally digest their food well." But we beg pardon for having so long detained our readers by the exposition of such absurdities. We feel it a sort of degradation that such a book as this *could* emanate from a titled member of our profession.

